# Inflammatory cytokines and biofilm production sustain *Staphylococcus aureus* outgrowth and persistence: a pivotal interplay in the pathogenesis of Atopic Dermatitis

**DOI:** 10.1038/s41598-018-27421-1

**Published:** 2018-06-28

**Authors:** E. G. Di Domenico, I. Cavallo, V. Bordignon, G. Prignano, I. Sperduti, A. Gurtner, E. Trento, L. Toma, F. Pimpinelli, B. Capitanio, F. Ensoli

**Affiliations:** 1 Clinical Pathology and Microbiology, San Gallicano Institute, Istituto di Ricovero e Cura a Carattere Scientifico (IRCCS), 00144 Rome, Italy; 2 Biostatistics, San Gallicano Institute, Istituto di Ricovero e Cura a Carattere Scientifico (IRCCS), Rome, Italy; 3 Department of Research, Advanced Diagnostics, and Technological Innovation, Translational Research Area, Regina Elena National Cancer Institute, Istituto di Ricovero e Cura a Carattere Scientifico (IRCCS), 00144 Rome, Italy; 4 Infectious Disease Consultant, Regina Elena National Cancer Institute, Istituto di Ricovero e Cura a Carattere Scientifico (IRCCS), 00144 Rome, Italy; 5 Division of Dermatology, San Gallicano Institute, Istituto di Ricovero e Cura a Carattere Scientifico (IRCCS), 00144 Rome, Italy

## Abstract

Individuals with Atopic dermatitis (AD) are highly susceptible to *Staphylococcus aureus* colonization. However, the mechanisms driving this process as well as the impact of *S. aureus* in AD pathogenesis are still incompletely understood. In this study, we analysed the role of biofilm in sustaining *S. aureus* chronic persistence and its impact on AD severity. Further we explored whether key inflammatory cytokines overexpressed in AD might provide a selective advantage to *S. aureus*. Results show that the strength of biofilm production by *S. aureus* correlated with the severity of the skin lesion, being significantly higher (P < 0.01) in patients with a more severe form of the disease as compared to those individuals with mild AD. Additionally, interleukin (IL)-β and interferon γ (IFN-γ), but not interleukin (IL)-6, induced a concentration-dependent increase of *S. aureus* growth. This effect was not observed with coagulase-negative staphylococci isolated from the skin of AD patients. These findings indicate that inflammatory cytokines such as IL1-β and IFN-γ, can selectively promote *S. aureus* outgrowth, thus subverting the composition of the healthy skin microbiome. Moreover, biofilm production by *S. aureus* plays a relevant role in further supporting chronic colonization and disease severity, while providing an increased tolerance to antimicrobials.

## Introduction

Atopic dermatitis (AD) is a chronic skin disease characterized by impaired epidermal barrier function, cutaneous inflammation and a high degree of *Staphylococcus aureus* colonization in the skin lesions. AD affects approximately 15–30% of the children^1,2^ with 60% of cases occurring within a child’s first year and 85% before the age of 5^3^. Mutations in the filament aggregating protein (filaggrin), which is encoded by the *FLG* gene, has been considered a major predisposing genetic element for AD^4–7^. Filaggrin is a natural moisturizing factor in the stratum corneum of the skin and mutations in the *FLG* gene may impair the skin barrier function^8–11^. However, the majority of patients with AD do not harbour any mutation in the *FLG* gene and approximately 60% of mutation carriers do not present any clinical evidence of AD. This indicates that *FLG* mutations are neither necessary nor sufficient to cause AD, suggesting that other mechanisms affecting the skin barrier integrity may play a part in AD pathogenesis^12–14^.

The clinical hallmark of AD is the cutaneous inflammation, characterised by increased epidermal thickness, enhanced expression of inflammatory cytokines and the presence of a dense infiltrate of activated CD4+T cells^15,16^. Based on the predominant expression of specific cytokines, the immune response in AD has been described to develop in two distinct phases, early (acute) and late (chronic), respectively^16,17^. The interleukin (IL) 1 super family is the major epidermal proinflammatory cytokine complex involved in acute inflammation^18^. Increased level of IL-1, particularly IL-1β, correlates with signs of clinical exacerbation and progression of AD^19,20^. The initiation of the acute inflammatory response is also triggered by an increased Th2 activity, characterised by the expression of IL-4, IL-5, IL-13, and by a reduced production of interferon (IFN) γ. Conversely, the chronic phase of inflammation associates with an increase of the Th1 response, with an overproduction of IL-12 as well as of IL-5, IL-8 and IFN-γ, which represent the typical markers of chronic skin inflammation^16^. Notably, the overproduction of Th2 cytokines has been shown to alter the expression of different terminal differentiation proteins encoded by keratinocytes, including filaggrin, thus directly contributing to cutaneous barrier function impairment^12,21,22^. In addition, Th2 cytokines can downregulate the skin antimicrobial peptides (AMP) such as the human beta defensin (HBD)-2, HBD-3, and LL-37, which play major roles at contrasting colonization/infection of the skin by pathogenic bacteria^23,24^. Accordingly, microbiome analyses in AD lesions revealed a drastic reduction of the skin microbial diversity, with a low frequency of coagulase-negative staphylococci (CoNS), and an overabundance of *S. aureus*^25,26^. More than 80% of patients with AD appear to be permanently colonized with *S. aureus* in the eczematous skin lesions and disease severity has been directly correlated to the degree of *S. aureus* colonization^22,25,27–30^.

Antibiotic treatment usually reduces or apparently eliminates *S aureus*, however frequent recolonization occurs within few weeks with limited clinical improvement^31,32^. The difficulty in eradicating *S. aureus* colonization with conventional antibiotic therapy may be evocative of the presence of biofilms. Indeed, *S. aureus* biofilms have been observed, as predominant species, in patients with AD lesions^33–36^.

The mechanisms governing *S. aureus* overabundance, its chronic persistence in the skin as well as bacterial factors capable of influencing AD clinical expression are as yet unclear.

In this study, we investigated whether biofilm production by *S. aureus* might play a part at sustaining *S. aureus* persistence and assessed its impact on AD severity. Besides, we evaluated whether biofilm production by *S. aureus* strains might impact antibiotic tolerance. Finally, we explored the possible relationship between the production of key inflammatory cytokines such as IL-1β, IL-6 and IFN-γ and the growth and adhesion capabilities of *S. aureus* strains isolated from patients with AD.

## Results

### *Staphylococcus* aureus colonization correlates with the severity of AD

From the 81 patients with AD, 42 were females (51.8%) and 39 (48.2%) males, aged between 3 months and 12 years. The overall prevalence of *S. aureus* on the lesional skin of AD patients was found to be 53.1% (44/81) (Table 1). In particular, *S. aureus* was isolated in 7 out of 25 (28.0%) patients classified with mild AD, in 17 out of 30 (56.6%) with moderate AD and 20 out of 26 (76.9%) with the severe form of AD (Table 1). Comparative analysis showed that *S. aureus* colonization was significantly lower in patients with mild AD as compared to individuals with moderate AD (P = 0.03) and to those with severe AD (P < 0.01). In contrast, the difference in the degree of *S. aureus* colonization in patients belonging to either the moderate or severe AD groups, was not significant (P = 0.07).

**Table 1:** *Staphylococcus aureus* prevalence in AD skin lesions.

SCORAD	N° of patients	Positive	Positive (%)
Mild	25	7	28.0
Moderate	30	17	56.6
Severe	26	20	76.9
Total	81	44	54.3

These data confirmed that an increased *S. aureus* colonization is associated with the severity of the skin lesion in AD.

### High Biofilm producing isolates are associated with severe forms of AD

Previous reports suggested that biofilm may represent a potential virulence determinant in AD^33,35,37^. To further address this issue, the presence of the intercellular adhesion genes (*ica*) *A* and *D* genes, which are specific biofilm-associated genes of the *ica* operon, was analyzed in all isolates. The results confirmed that all the strains were positive for both *icaA* and *icaD* genes suggesting that the *S. aureus* isolates have the potential to produce biofilm (Supplementary Table 1).

**Figure 1:**
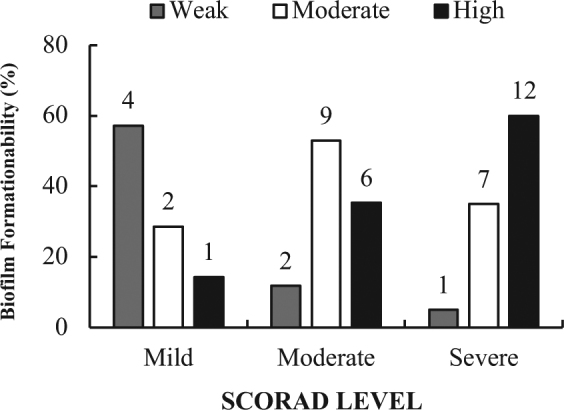
Biofilm production by *Staphylococcus aureus* isolates. Biofilm formation was assessed in the 44*S. aureus* strains isolated from patients with different degrees of AD. The severity of AD was classified as Mild, Moderate and Severe, respectively, according to the ‘scoring atopic dermatitis’ index (SCORAD). The values above the bars show the the exact number of biofilm-forming isolates. The level of biofilm production was measured by the clinical Biofilm Ring Test (cBRT) and the isolates classified as weak, moderate and high biofilm producers, respectively.

To confirm the results of the genetic analysis and to further evaluate the phenotypic expression and quantify the levels of biofilm production, all the *S. aureus* strains were tested by the clinical Biofilm Ring Test® (cBRT)^38^. The results showed that all isolates were able to produce biofilm, confirming that the simultaneous positivity to the *icaA* and *icaD* genes is always associated with the presence of biofilm-producing *S. aureus*^35,39,40^. In particular, 43.0% of *S. aureus* strains were classified as high biofilm producers, 41.0% as moderate and 16.0% were identified as weak biofilm producers. By comparing the degree of severity of the skin lesions with the ability to produce biofilm emerged that 60.0% of patients with severe AD were colonized by high biofilm producers, 35.0% by moderate biofilm producers and only 5.0% by weak biofilm producers. In contrast, 57.1% of patients with a mild AD form were colonized by weak biofilm producers and only in 14.3% of them the colonization was sustained by high biofilm producers (Fig. 1). Statistical analysis showed that patients with severe AD had a significantly higher probability (P < 0.01) to be colonized by strong biofilm producer *S. aureus* strains as compared with patients with mild AD.

**Figure 2:**
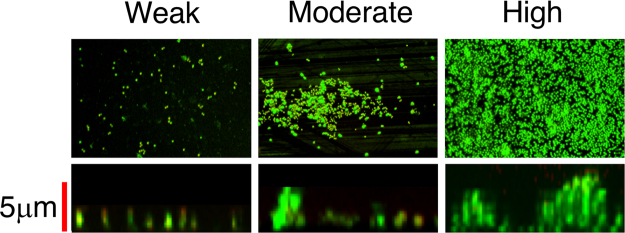
Confocal microscopy images of biofilms. Representative images of biofilms developed on polystyrene pegs following 24 h incubation at 37 °C. *Staphylococcus aureus* isolates are classified into three groups (weak, moderate and high) according to their biofilm-forming ability. Upper panels show the X-Y planes (top view), while the lower panels show the Z section (side view).

Confocal microscopy analysis provided results in good accordance with those gathered by the cBRT (Fig. 2). The strains identified as weak biofilm producer formed small cellular clumps on the surfaces of a polystyrene slide with a thin three-dimensional microcolony structures, the moderate biofilm producer developed much bigger clumps, whereas high biofilm producer exhibited a full coverage of the slide with the development of a thick and more structurally complex biofilm.

### Antimicrobial susceptibility and identification of oxacillin-resistant *S. aureus* in patients with AD

**Figure 3:**
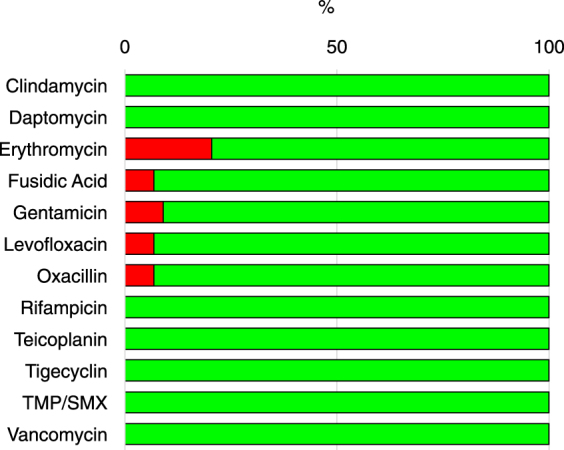
Antimicrobial susceptibility testing for the 44 *Staphylococcus aureus* isolates. Percentage of susceptible (green) and resistant (red) strains to the indicated antimicrobials, as determined by broth micro-dilution testing. Classification was performed according to the European Committee on Antimicrobial Susceptibility Testing clinical breakpoint tables (EUCAST Clinical Breakpoint Table v 7.1). TXP/SMX - Trimethoprim/Sulfamethoxazole

The conventional antimicrobial susceptibility testing, performed in planktonic microbial growth, with all the *S. aureus* strains showed a high level of susceptibility to most antimicrobial agents (Fig. 3). However, 77.2% of the isolates were resistant to benzylpenicillin, 20.4% to erythromycin, 15.9% to clindamycin and 9.1% to gentamycin. Moreover, three strains were found resistant to oxacillin (6.8%). Further analysis of these three strains confirmed their resistance profiles showing the presence of the *mecA* gene, which confers resistance to methicillin, and the presence of penicillin binding protein (PBP2), as assessed by the latex agglutination test. Taken together these data indicate a very low prevalence of MRSA strains. Further, statistical analysis confirmed that the oxacillin resistance profile was not associated with a significant increase in the severity of AD (P = 0.36).

### *In vitro* analysis of antibiotic susceptibility of biofilm-producing *Staphylococcus aureus*

**Figure 4:**
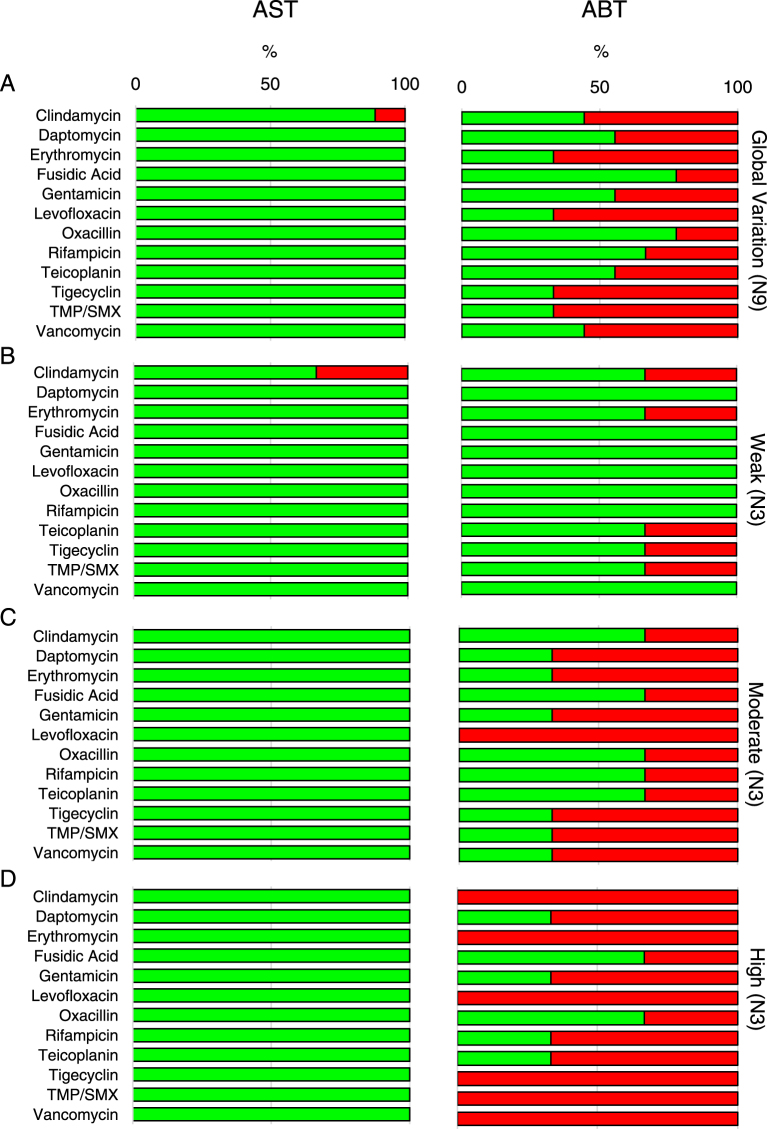
Comparison between the Antimicrobial susceptibility test (AST) and the Anti-Biofilm Test (ABT). (**A**) Overall percentage of susceptible (green) and resistant (red) *Staphylococcus aureus* strains to different antimicrobials as determined in the nine strains (N9). Comparison between AST and ABT in Weak (**B**), Moderate (**C**) and High (**D**) biofilm producing *Staphylococcus aureus* isolates. Classification was performed according to the European Committee on Antimicrobial Susceptibility Testing clinical breakpoint tables (EUCAST Clinical Breakpoint Table v 7.1). In brackets the number of strains analyzed. TXP/SMX - Trimethoprim/Sulfamethoxazole.

It has been reported that the use of anti-*Staphylococcus* agents in the treatment of AD has a reduced efficacy with a high rate of re-colonization occurring after 4–8 weeks^31^. These data are apparently in contrast with the susceptibility profiles observed in this study and those previously reported^32,41–43^. Thus, we evaluated whether the ability to produce biofilm might sustain an increased antibiotic tolerance, which, in turn, may contribute to re-colonization. These studies were performed in 9 clinical isolates, equally divided in weak, moderate and strong biofilm producers as described in Fig. 1. To ensure comparability of the data, the conventional Antimicrobial Susceptibility Testing (AST) and the Anti-Biofilm Test (ABT) were both performed by a broth dilution-based procedure, testing 12 antibiotics. AST performed on the 9 isolates, gave similar resistance profiles with only one strain, within the group of the high biofilm producers, found resistant to clindamycin (Fig. 4A). Conversely, the antibiotic susceptibility of *S. aureus* strains growing in mature biofilms, evaluated by ABT, gave resistance profiles which greatly differed (49.1% of the isolates) from those gathered by AST (Fig. 4B). By ABT testing, the most effective antibiotics were found to be fusidic acid and oxacillin, with a minimal biofilm-eradication concentration (MBEC) value below breakpoints in 77.8% of isolates. Rifampicin was effective in 66.7% of isolates and daptomycin, gentamicin and teicoplanin in 55.6% of them. On the contrary, erythromycin, levofloxacin, tigecycline and trimethoprim/sulfamethoxazole were the less effective antibiotics, showing a MBEC below breakpoints in only 33.3% of isolates. The spectrum of antimicrobial efficacy was found to be further restricted when the analysis was focused on moderate/high biofilm producer *S. aureus* strains. With all these strains levofloxacin was totally ineffective while erythromycin, tigecycline, trimethoprim-sulfamethoxazole and vancomycin gave a MBEC below breakpoints in only 16.6% of the strains. Interestingly, the comparative assessment of susceptibility profiles gathered by AST and ABT considering separately the Weak, Moderate and High biofilm producer groups, revealed differences of 11.1%, 55.6% and 77.8%, respectively. Statistical analysis confirmed significant differences of the antimicrobial susceptibility as assessed by AST and ABT in Weak (P = 0.01), Moderate (P < 0.001) and High (P < 0.001) biofilm producers, respectively. Further, ABT assessment showed also significant differences between Weak and both Moderate (P < 0.001) and High (P < 0.001) biofilm producers as well as between Moderate and High (P = 0.002) biofilm producers.

### Cytokine-induced growth of *S. aureus*

The previous set of studies indicated that the presence of *S. aureus* correlates with disease progression and that microbial biofilm-production may allow for increased adhesion and antibiotic tolerance, thus favoring an effective colonization and chronic persistence. Next, we sought to determine whether *S. aureus* colonization might take advantage from the inflammatory cytokine milieu which characterize the disease. To address this issue, we investigated whether IL-1β, IL-6 and IFN-γ, which are the major cytokines involved in the AD inflammatory response, were capable to support the growth of either planktonic or biofilm growing *S. aureus* strains.

**Figure 5:**
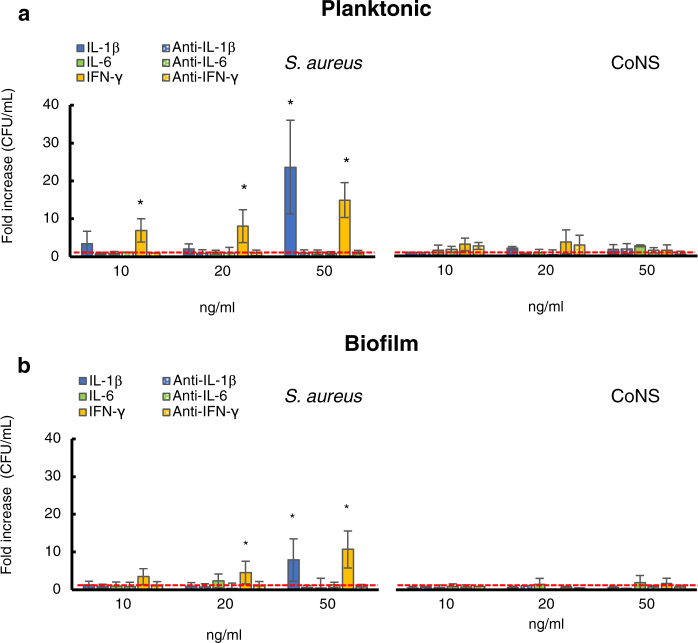
Growth response of *Staphylococcus aureus* isolates and coagulase-negative staphylococci (CoNS) to different concentrations of cytokines. Planktonic (panel A) and biofilm (panel B) bacteria were cultured overnight in RPMI medium alone (untreated) and in the presence of different concentrations of IL1-β, IL-6 and IFN-γ. Data are expressed as fold increase (CFU/mL) between treated and untreated cells (dashed red line). The strains were tested in triplicate and experiments were repeated at least three times for each cytokine concentration. Mann-Whitney nonparametric test was used for statistical analysis. *Denotes P < 0.05.

The initial inoculum for the *S. aureus* strains was 1.84 × 10^5^ ± 5.13 × 10^4^ CFU/ml. The bacterial counts after 24 h of incubation in RPMI, including both planktonic and biofilm bacteria, was 9.05 × 10^8^ ± 4.34 × 10^8^, which gave a mean generation time of 118 minutes. The growth of *S. aureus* strains, measured as CFU/mL, was assessed in the presence of different concentrations (50, 20, 10 ng/mL) of IL-1β, IL-6 and IFN-γ, respectively. The results of these experiments, which are summarized in Fig. 5A,B, showed a concentrations dependent, significant growth increase of *S. aureus* isolates in the presence of IL-1β and IFN-γ but not with IL-6, after 24 hours of incubation. This effect was observed with both planktonic and biofilm cultures (Fig. 5A,B). Specifically, treatment with IL-1β at 50 ng/ml gave a significant (P = 0.002) difference of bacterial count in planktonic and biofilm growth conditions, respectively, giving rise to a 23.6- and 7.9-fold increase. Conversely, no significant difference was observed with IL-1β at 10 and 20 ng/ml concentrations in both planktonic and biofilm cultures. In the presence of IFN-γ, *S. aureus* exhibited a concentration-dependent, significant increase of bacterial counts in planktonic cultures at 10 (P = 0.04), 20 (P = 0.03) and 50 ng/mL (P = 0.001), respectively, with bacterial counts peaking at up to 14-folds (Fig. 5A). Similarly, IFN-γ exerted significant growth-promoting activities on biofilms at 20 (P = 0.03) and 50 ng/mL (P = 0.006), respectively (Fig. 5B).

On the other hand, IL-6 did not show any significant growth increase of both planktonic and biofilm growing *S. aureus* strains after 24 hours of incubation, even at high cytokine concentrations (Fig. 5A,B).

The specificity of the growth-promoting activities of IL-1β and IFN-γ on *S. aureus* was further confirmed by neutralization experiments. The addition of anti-IL-1β and anti-IFN-γ neutralizing antibodies to the growth medium abolished the bacterial growth enhancement induced by either cytokine (Fig. 5A,B).

### Pro-inflammatory cytokines fail to stimulate the growth of coagulase-negative staphylococci

*S. epidermidis* and *S. hominis* are common skin commensals, representing the most abundant Coagulase negative staphylococcal species (CoNS) populating the skin microbiome^44^. Since CoNS and *S. aureus* share the same habitat on the human skin, we evaluated whether the growth-promoting activities of inflammatory cytokines were selectively exerted on *S. aureus* or might also include other skin commensals such as *S. epidermidis* and *S. hominis*. The initial inoculum for CoNS strains was 9.25 ± 3.77 × 10^4^ CFU/ml. The bacterial counts after 24 h of incubation in RPMI, was 9.40 × 10^8^ ± 4.18 × 10^8^ CFU/ml corresponding to a mean generation time of 127 minutes, which is remarkably similar to the mean generation time measured for *S. aureus* isolates. The growth of CoNS strains, in the presence of different concentrations of IL-1β, IL-6 and IFN-γ, showed that none of the cytokines tested had any effect on CoNS isolates in both planktonic and biofilm cultures (Fig. 5A,B). These data suggest that the ability to respond to cytokines may not represent a general property of staphylococci, but it is rather related to the acquisition of a specie-specific competence which confers a selective advantage to *S. aureus* strains with respect to other skin commensals.

## Discussion

AD is the most common chronic inflammatory skin disease in the general population^45^. The pathogenesis of AD appears to be the result of a complex interplay between skin barrier defects and altered immune response^22,46^. The skin of individuals with AD is particularly vulnerable to colonization with *S. aureus* and an increasing body of evidence suggest that its presence correlates with skin inflammation and disease severity^22,47^. In agreement with previous reports we found that AD skin lesions appear particularly predisposed to *S. aureus* colonization. Specifically, of 81 patients with AD included in the study, 44 (54.3%) were colonized by *S. aureus*, with a different distribution according to the severity of the disease. *S. aureus* was present in 28.0%, 56.6% and 76.9% of patients with mild, moderate and severe forms of AD, respectively. Indeed, colonization with *S. aureus* was significantly higher in patients with moderate (P = 0.03) or severe (P < 0.01) AD than in individuals with mild disease forms. Prevalence analysis showed rates of *S. aureus* colonization of 75~90% in the lesional skin in AD, whereas in healthy individuals this bacterium is present only in 5~30% of cases, according to different studies^33,48–50^. The overall prevalence of *S. aureus* (53.1%) is lower than that previously reported, however, this apparent discrepancy can be explained by the inclusion in our study of patients with moderate and mild forms of AD. Indeed, the prevalence of *S. aureus* in patients with severe AD peaks at 76.9%, which is in line with previously reported values.

We found that the majority of *S. aureus* strains isolated from AD lesions can produce several toxins and superantigens, which are thought to play an important role in the natural course of AD^45,51–53^. However, we did not find any direct relationship between the presence of a specific toxin gene and the severity of AD (Data not shown).

The mechanisms allowing *S. aureus* to preferentially colonize AD skin lesions are still unclear. The molecular analysis aimed at assessing the presence of genes coding for biofilm components showed that all isolates harbored both *icaA* and *icaD* genes. This is in agreement with previous studies reporting that 92.5% of *S. aureus* strains isolated from AD patients were positive for both *icaA* and *icaD* genes^35^. To evaluate the interplay between *S. aureus* adhesion capabilities and the severity of AD, the phenotypic expression and quantitative production of biofilm was further examined by the cBRT^38^. We found that the ability to form biofilm by *S. aureus* isolates was significantly associated (P < 0.01) with the severity of the disease. Indeed, patients with more severe form of AD were invariably colonized by strong biofilm producers. This data suggest that biofilm represents a key factor for *S. aureus* persistent colonization in the eczematous skin of AD by enhancing adhesion and providing protection from the host immune response, competitor microbial species as well as antimicrobials^35^. This notion is indirectly reinforced by the results of confocal microscopy, which clearly shows the development of a thick, highly adhesive and structurally complex biofilm matrix, capable of exerting a remarkable barrier effect against multiple biologic and chemical insults.

The correlation between the overabundance of *S. aureus* and AD exacerbation, provides some support to the use of antibiotics for AD treatment. Although antibiotics can reduce or apparently eliminate *S. aureus*, recolonization frequently occurs within few weeks, with limited clinical efficacy^31,32^. Such a capability of chronic persistence and relapse following antibiotic therapy is strongly suggestive of a biofilm-related colonization, which may explain the high tolerance of *S. aureus* to antimicrobial treatment^31,32,41–43,54,55^ as well as the conflicting evidence about the clinical benefit of the use of anti-staphylococcal agents^31,32,56–59^, even in those cases in which therapy was based on MIC results^60^. This does not appear to depend on the expression of multi drug resistance genes, since a very low prevalence of MRSA in AD has been previously reported^41–43,61–63^ and was found in the present study with only 6.8% of the isolates classified as MRSA.

The major problem in treating biofilm-related infections is due to the higher concentration of antibiotics required to kill bacteria, which can be hundred times higher than the MIC for the same microorganism as assessed by conventional AST performed in planktonic culture^64–66^. In our study, biofilm-growing cultures were found to be much more tolerant to antibiotics as compared to planktonic-growing cultures. The antibiotic susceptibility assessment by AST, revealed similar profiles among the different isolates (Fig. 4A). However, biofilm growing isolates showed increased levels of antibiotic tolerance as compared to their planktonic counterparts with significant differences among Weak (P = 0.01), Moderate (P < 0.001) and High (P < 0.001) biofilm producers, respectively (Fig. 4). In particular, biofilm production led to significant differences between Weak and both Moderate (P < 0.001) and High (P < 0.001) biofilm producers as well as between Moderate and High (P = 0.002) biofilm producers, respectively. Thus, tolerance of *S. aureus* isolates to antibiotics was directly related to the quantitative levels of biofilm production. Interestingly, we found that fusidic acid and oxacillin were the most effective molecules against biofilm-producing strains, with an MBEC below the threshold in approximately 80% of isolates. Further, our data showed that rifampin was active against biofilm-producing *S. aureus* with a MBEC below the threshold in 66.7% of isolates and this evidence is also consistent with previous studies reporting rifampin as an active compound against biofilm^67^.

Several studies have shown that local use of corticosteroids, even in the absence of antibiotics, reduces the colonization of *S. aureus* through an undefined process^49,68–71^.

Since corticosteroids mainly play an anti-inflammatory and immunosuppressive action, it is conceivable that cytokines involved in the inflammatory response may play a role in promoting *S. aureus* colonization. Thus, we evaluated the growth promoting activity of the prototypical cytokines involved in the acute (IL-1β, IL-6) and chronic (IFN-γ) phases of the disease on *S. aureus* strains isolated from AD patients with mild, moderate and severe forms of the disease, respectively. The cytokine concentrations we assessed have been previously tested and considered physiologically relevant in AD^12,72–74^. The results showed that IL-1β and IFN-γ but not IL-6 induce a significant growth enhancement on both planktonic- and biofilm-growing *S. aureus* strains in a concentrations dependent manner (Fig. 5). These results indicate that both early and late AD inflammatory cytokines are capable of supporting *S. aureus* planktonic and biofilm outgrowth during both the acute and chronic phases of the disease. These data further confirm earlier evidence of the growth promoting potential of IL-1β on *S. aureus*^72,74,75^. However, our data, which were gathered by testing field-isolated strains, show that IL-1β can stimulate the growth of both planktonic and biofilm cultures, while previous reports, dealing with reference laboratory strains, showed a growth enhancement for only the biofilm component. This is a relevant issue, since planktonic and biofilm microbial cells coexist in the same environment *in vivo*. The lack of growth-induction activity of inflammatory cytokines on other bacterial component of the skin microbiome suggests the existence of an adaptive response, specifically evolved by *S. aureus* and not present on CoNS, to take advantage of eukaryotic epigenetic signals. This selectivity provide support to *S. aureus* outgrowth at the expenses of other microbial commensals, further favoring *S. aureus* persistence. Indeed, these bacteria also produce potent and strain-specific AMPs which selectively kill *S. aureus*. A low frequency of these species correlates with increased colonization by *S. aureus*, further reinforcing the notion that commensal skin bacteria have a protective role against pathogens^25,26^.

Since microbial biofilm is generally associated with disease chronicity, the response of *S. aureus* to IFN-γ may play a key role on AD disease severity. Indeed, previous reports showed that murine models defective for the Th1 response were capable to clarify *S. aureus* from their nose more efficiently than their wild type counterpart and that the presence of IFN-γ played a detrimental role in murine defense against nasal colonization of slime producing *S. aureus* ATCC 35556^76^. Regarding the lack of growth promoting activity by IL6, previous studies showed that IL-6 enhanced the growth of fresh *S. aureus* isolates while this ability was lost in strains which underwent at least six plate passages^72^. Our isolates generally underwent at least 3/4 plate passages before the setting of cytokine cultures, thus we cannot exclude that this procedure caused the absence of a growth response to IL-6.

The molecular mechanism responsible for the growth response of *S. aureus* to cytokines remains to be defined, also considering that in eukaryotic cells the transduction of cytokine signals follows specific pathways which are putatively absent in bacteria. Nevertheless, cytokine-induced bacterial growth has been also shown in *Acinetobacter* spp. and *Pseudomonas aeruginosa*, while high levels of cytokines production correlates with increased severity and mortality in critically ill patients harboring bacterial infections^72,77,78^. Interestingly, a methyl-accepting chemotaxis proteins (MCPs), including five chemoreceptors, has been recently described in *Escherichia coli*, having the potential to mediate growth enhancement in the presence of IL-2, granulocyte-macrophage colony-stimulating factor (GM-CSF) IL-15, and TNF-α^79–82^. The surface of Gram-negative bacteria has chemoreceptors responding to proinflammatory cytokines that have the potential to alter the microbial virulence properties^82–84^. In particular, TNF-α and IFN-γ induce *E. coli* translocation in the gut and IL-8 promote transmigration across human lung epithelium in pulmonary infections suggesting that inflammatory cytokines represent specific chemoattractants for bacterial translocation^82–84^. *S. aureus* might take advantage from a signalling process similar to that described for *E. coli* and involving biochemical pathways, other than those of the eukaryotic counterparts, capable of transducing the signal across the bacterial cell membrane and inducing gene modulation.

**Figure 6:**
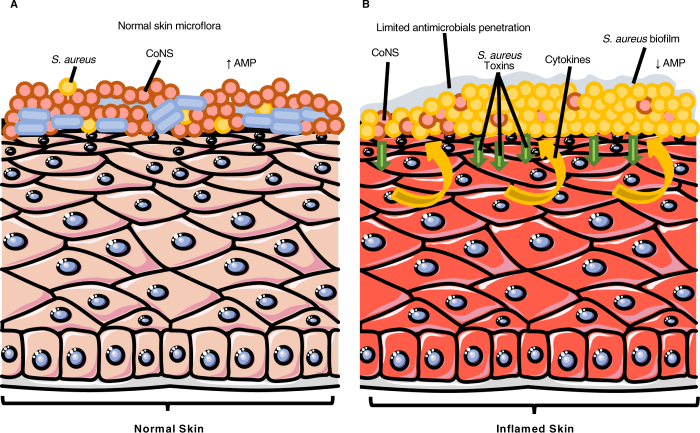
Schematic illustration of the pathophysiological changes between normal (**A**) and inflamed skin in patient with atopic dermatitis (**B**). Arrows indicate increase (↑) and decrease (↓). The epithelium (https://smart.servier.com/smart_image/epithelium-17/) is adapted from Servier Medical Art (http://smart.servier.com) under the Creative Commons License.

Although further research is needed to elucidate these mechanisms, our results suggest that specific cytokines produced during the acute and chronic AD phases can promote S. aureus outgrowth at the expenses of other microbial components of the skin microbiome and that microbial biofilm production can contribute at supporting bacterial adhesion, chronic persistence and increased tolerance to antimicrobials. It is tempting to speculate that the advantage provided by biofilm in promoting a persistent colonization, may also lead to a chronic release of toxins and superantigens, that, in turn, may either directly or indirectly contribute to the severity of AD. This might trigger a vicious circle that causes further barrier damages and a subsequent release of cytokines that additionally support *S. aureus* growth and chronic persistence (Fig. 6).

Thus, an interplay between host (inflammatory cytokines) and bacterial (biofilm production) factors may play pivotal roles in the pathogenesis of AD. This notion further supports the use of corticosteroids for the treatment of AD, for their anti-inflammatory action and the ensuing expected impact on *S. aureus* colonization. The association of antimicrobials should be carefully evaluated on the basis of the assessment of their efficacy against bacterial biofilm.

## Materials and Methods

### Ethics Approval

The study was approved by the Central Ethics Committee I.R.C.C.S. Lazio, section of the Istituti Fisioterapici Ospitalieri, Rome (Prot. CE/1016/15—15 December 2015, trials registry N. 730/15). All the procedures and methods have been carried out in accordance with the guidelines laid down by the Ethics Committee and in accordance with local laws and regulations. Informed consent was obtained from all subjects.

### Patients

Sample collection was performed on a total of 81 AD patients attending the Pediatric Outpatient Clinic at the San Gallicano Dermatology Institute of Rome, Italy from December 2015 to January 2017. There was no selection of patient’s gender, or severity of lesions. The study group comprised children aged between 3 months and 12 years, of both sexes (42 females and 39 males). All individuals were subjected to a complete clinical history followed by clinical examination. The clinical severity of AD was determined according to the ‘scoring atopic dermatitis’ index (SCORAD), as described by Hanifin and Rajka^85^. Accordingly, the clinical expression of disease was classified as mild, moderate, or severe according to SCORAD values ranging between 0 and 15, 15 and 40 or >40, respectively^33^. Patients presenting with fungal infection or other skin diseases were excluded from the study, as well as those that received any steroid or antibiotic therapy in the last two months before study initiation.

Samples were collected by commercially available sterile swabs (COPAN swabs, Brescia, Italy), according to existing departmental guidelines. The samples were sent to the laboratory and processed within 30 minutes after collection for culture analysis.

### Bacterial identification from skin samples

The swabs were plated onto blood agar, Mannitol Salt Agar (MSA) and *S. aureus* ID Agar (SAID) plates (bioMérieux, Bagno a Ripoli, Italy) for the initial identification of *S. aureus* and onto MacConkey agar and Sabouraud Agar (bioMérieux, Bagno a Ripoli, Italy) to verify the absence of Gram-negative bacteria and fungal infections. Inoculated plates were incubated aerobically for 24 hours at 37 °C. The automated VITEK® 2 system (bioMérieux, Bagno a Ripoli, Italia) was used for the subsequent bacterial identification. Further microbial identification was performed by matrix assisted laser desorption/ionisation – time of flight mass spectrometry (MALDI-TOF MS system – Bruker Daltonics, Bremen, Germany)^86^. Antimicrobial susceptibility testing (AST) was performed by the broth microdilution test (Thermo Scientific, Massachusetts, USA) for the definition of the Minimum Inhibitory Concentration (MIC) criteria, according to the European Committee on Antimicrobial Susceptibility Testing (EUCAST Clinical Breakpoint Table v 7.1). The range of antibiotics tested is listed in the supplementary material section (Supplementary Table 2). Strains were classified as MRSA when presenting both oxacillin resistance (MIC ≥ 4 mg/ml) and positive agglutination test for Penicillin-Binding Protein (PBP2, Oxoid, Basingstoke, UK)^87^. *S. aureus, S. epidermidis* and *S. hominis* strains were frozen and stored in CRYOBANK (Copan, Brescia, Italia) at −80 °C.

### Molecular characterization of *Staphylococcus aureus* virulence genes

PCR assay was used for the detection of *S. aureus* virulence genes among isolates. The NucliSENS® easyMAG automatic extractor (bioMèrieux, Florence, Italy) was used for total nucleic acid extraction from *S. aureus* isolates. Detection of the 16 S rRNA gene was included as an internal positive control^86^. Reference strains from the American Type Culture Collection (ATCC): *S. aureus* ATCC 25923, *S. aureus* ATCC 6538, *S. epidermidis* ATCC 14990 and *S. epidermidis* ATCC 12228 were used as positive controls. The total bacterial DNA was amplified by PCR to assess the presence of the intracellular adhesion (*ica*) genes *A* and *D*^35,39,40^ and the gene for methicillin resistance *mecA*^88,89^. A complete list of primers is provided in Supplementary Table 3.

### Evaluation of *Staphylococcus aureus* biofilm production

Biofilm production by the *S. aureus* isolates was quantified by the clinical BioFilm Ring Test (cBRT) as previously described^38,87^. The test was performed using the reagents and equipment provided by the Biofilm Ring Test® kit (KITC004) and analysed by the BFC Elements 3.0 software (Biofilm Control, Saint Beauzire, France)^90^. Specifically, two *S. aureus* reference strains (ATCC 25923 and ATCC 6538) were included in each plate as standard reference and quality control. A well containing the BHI/TON mix without microbial cells was used as negative control in each experiment.

Each *S. aureus* strain was analysed in duplicate and experiments were repeated 3 times.

### Biofilm Imaging

*S. aureus* colonies, grown overnight on blood agar plates, were used to inoculate 3 ml of 0.45% saline solution (Air Life, Carefusion, CA, USA) to obtain a turbidity of 0.5 ± 0.1 McFarland (McF) corresponding approximately to 1 × 10^8^ colony-forming units (CFU)/ml. Samples were diluted 1:1000 and resuspended in 3 ml of BHI containing sterile polystyrene slices of 1 cm^2^. Bacterial suspension was incubated at 37 °C for 22 hours to allow biofilm formation onto polystyrene slices. Subsequently, the medium was removed and the polystyrene slices were washed in 0.45% saline solution. The samples were stained using the LIVE/DEAD BacLight kit (Life Technologies, New York, NY, USA), according to supplier specifications. Images were collected by a Zeiss LSM 510 Meta confocal laser scanning microscope equipped with a 20X and 60X/1.23 NA oil immersion objective (Carl Zeiss, Jena, Germany). Data were analyzed with the LSM 510 R. 3.2 META image analysis software (Carl Zeiss, Jena, Germany).

### Anti-biofilm drug susceptibility testing

The anti-biofilm test (ABT) was performed by the protocol described in Chronopoulou *et al*. with some modifications^91^. Nine *S. aureus* strains, including three weak, three moderate and three strong biofilm producers, respectively, were collected from a blood agar plate and inoculated into 2 ml of 0.45% saline solution (Air Life, Carefusion, CA, USA) to obtain a turbidity of 0.5 ± 0.1 McF. Samples were diluted 1:100 in BHI and 100 μl of bacterial suspension, corresponding approximatively to 1 × 10^6^ CFU/ml, were seeded into a sterile 96-multiwell polystyrene plate (Corning Inc., Corning, NY, USA). The plate was incubated at 37 °C for 22 hours to allow biofilm formation. Subsequently, medium was removed and the wells were washed twice with 100 μl of sterile distilled water to remove non-adherent cells. The preformed bacterial biofilm was incubated for additional 22 hours in 100 μl of BHI in the presence of different antibiotics at predefined concentrations (See supplementary material). After overnight treatment, antibiotics were removed, the plate washed twice with 200 μl of sterile distilled water and air-dried for 15 minutes at room temperature. Thereafter, the bottom of the wells was stained by 200 μl of a 0.1% violet crystal (CV) solution. After 20 minutes, the plate was washed two times with 300 μl of sterile distilled water to remove excess stain. The CV incorporated by the cells within the biofilm was dissolved in 200 μl of ethanol/acetone 80/20% and the optical density (OD) for each well was measured at 570 nm using the PhD™ lx System (Bio-Rad Laboratories, Hercules, CA, USA). Each plate included 10 wells without bacteria as negative control and 3 wells plated with bacteria in the absence of antibiotics as positive controls. The cut-off value has been defined as three standard deviations above the mean OD_570_ value of the negative controls. OD_570_ values above the threshold value identified bacterial growth and hence were used to identify the minimal biofilm-eradication concentration (MBEC), with reference to the EUCAST Clinical Breakpoint Table v 7.1 to identify the specific antimicrobial susceptibility.

### Assessment of bacterial growth in the presence of cytokines

To determine the effects of cytokines on bacterial growth, a sterile 96-multiwell polystyrene plate was plated with 200 μl of an initial bacterial suspension of 1 × 10^5^ CFU/ml in RPMI containing 2mM L-glutamine and 4 mM sodium pyruvate (EuroClone spa, Milan, Italy). Human cytokines interleukin (IL)−1β, IL-6 and IFN-γ (PeproTech, Rocky Hill, NJ, USA) were added to the culture medium at different concentrations (10 ng/ml, 20 ng/ml and 50 ng/ml). Since cytokines were lyophilized in the presence of bovine serum albumin (BSA), a 0.1% solution of BSA (Sigma-Aldrich, St. Louis, MI, USA) was added in the wells containing the negative controls^72^. Bacterial cultures were incubated at 37 °C for 24 hours without shaking^74^. Subsequently, planktonic cells were collected with the supernatant and the wells were washed twice with 0.45% saline solution. To measure the number of the adherent cells remaining in the wells, bacteria were detached by scraping the bottom of the wells with a pipet tip and resuspended in 200 μL of sterile distilled water. Serial 10-fold dilutions of both planktonic and adherent bacterial cells were prepared and 5 μLwere spotted on blood agar plates (bioMérieux, Bagno a Ripoli, Italy). Plates were incubated at 37 °C for 24 hours and then counted to calculate the CFU/ml^38^. For the *S. aureus* experiments six strains were selected including two weak, two moderate and two strong biofilm producers, respectively. For CoNS, three *S. epidermidis* and three *S. hominis* isolates collected from the skin of AD patients were tested.

Blocking experiments were performed by adding the corresponding neutralizing monoclonal antibody in the culture medium, following manufacturer’s instruction (PeproTech, Rocky Hill, NJ, USA). Cytokines and neutralizing antibodies were incubated at 4 °C for one hour and then added to the growth culture medium. Subsequently, a bacterial suspension of 1 × 10^5^ CFU/ml was added to each well and incubated at 37 °C for 24 hours. Planktonic and biofilm bacterial cells were counted as described above.

The bacterial growth rates and the mean generation time were calculated using the following equation:

1 $${\mathrm{log}}_{10}{\rm{Nt}}={\mathrm{log}}_{10}{{\rm{N}}}_{0}+{\rm{g}}\,{\mathrm{log}}_{10}2$$

where N_t_ is the number of cells after 24 h of incubation in RPMI and N_0_ is the number of cells at time zero.

Each strain was tested in triplicate and experiments were repeated at least three times for each cytokine concentration.

### Statistics

Descriptive statistics were used to summarize pertinent study information. The association between SCORAD level and biofilm production was tested by chi-square test for linear trend. The Mann-Whitney nonparametric test was used to compare quantitative variables.

p-values of 0.05 or less were considered statistical significant. IBM SPSS v.21 statistics software (IBM, Chicago, IL, USA) was used for all statistical analyses.

## Electronic supplementary material

Inflammatory cytokines and biofilm production sustain Staphylococcus aureus outgrowth and persistence: a pivotal interplay in the pathogenesis of Atopic Dermatitis
