# Supplementary material for: Inflammatory cytokines and biofilm production sustain Staphylococcus aureus outgrowth and persistence: a pivotal interplay in the pathogenesis of Atopic Dermatitis
Source: Sci Rep. 2018 Jun 28;8:9573. doi: 10.1038/s41598-018-27421-1 (PMC6023932; doi:10.1038/s41598-018-27421-1)
Supplement: Supplementary file 1 — Inflammatory cytokines and biofilm production sustain Staphylococcus aureus outgrowth and persistence: a pivotal interplay in the pathogenesis of Atopic Dermatitis [file 41598_2018_27421_MOESM1_ESM.docx]

**Inflammatory cytokines and biofilm production sustain *Staphylococcus aureus* outgrowth and persistence: a pivotal interplay in the pathogenesis of Atopic Dermatitis**

E. G. Di Domenico, I. Cavallo, V. Bordignon, G. Prignano, I. Sperduti, A. Gurtner, E. Trento, L. Toma, F. Pimpinelli, B. Capitanio & F. Ensoli

**Supplementary Material**

| Genes | SCORAD | | |
| --- | --- | --- | --- |
|  | Mild (N7) | Moderate (N17) | Severe (N20) |
| *icaA* | 7 | 17 | 20 |
| *icaD* | 7 | 17 | 20 |

Supplementary Table 1. Prevalence of intercellular adhesion genes (*ica*) *A* and *D*.

N refers to the number of strains analysed for each group.

Supplementary Table 2**.** List and concentration range (μg/ml) of the antibiotics tested.

| Antibiotic | Conc. Range (μg/ml) |
| --- | --- |
| Clindamycin | 0.125 - 1 |
| Daptomycin | 0-25 - 4 |
| Erythromicin | 1 - 4 |
| Fusidic Acid | 0.125 - 1 |
| Gentamicin | 0.125 - 4 |
| Levofloxacin | 0.125 - 4 |
| Oxacillin | 0.25 - 2 |
| Rifampicin | 0.0625 - 2 |
| Teicoplanin | 0.25 - 4 |
| Tigecycline | 0.0625 - 0.5 |
| TMP/SMX | 0.25/4.75 - 4/76 |
| Vancomycin | 0.5 - 4 |

**TXP/SMX - Trimethoprim/Sulfamethoxazole**

| Primer | Sequence (5’-3’) | Reference |
| --- | --- | --- |
| *icaA*-F | TCTCTTGCAGGAGCAATCAA | Gad et al., 2009 |
| *icaA*-R | TCAGGCACTAACATCCAGCA |  |
| *icaD*-F | ATGGTCAAGCCCCAGACAGAG | Gad et al., 2009 |
| *icaD*-R | CGTGTTTTCAACATTTAATGCAA |  |
| *mecA*-F | ACTGCTATCCACCCTCAAAC | Mehrotra et al., 2000 |
| *mecA*-R | CTGGTGAAGTTGTAATCTGG |  |
| *16SrRNA*-F | GAGAGTTTGATCCTGGCTCAG | Di Domenico et al., 2015 |
| *16SrRNA*-R | CTACGGCTACCTTGTTACGA |  |

Supplementary Table 3. List of primers used in this study
